# Distributional interaction: Interpretational problems when using incidence odds ratios to assess interaction

**DOI:** 10.1186/1742-5573-2-1

**Published:** 2005-03-03

**Authors:** Ulka B Campbell, Nicolle M Gatto, Sharon Schwartz

**Affiliations:** 1 Department of Epidemiology, Mailman School of Public Health at Columbia University, New York, USA

## Abstract

It is well known that the incidence odds ratio approximates the risk ratio when the disease of interest is rare, but increasingly overestimates the risk ratio as the disease becomes more common. However when assessing interaction, incidence odds ratios may not approximate risk ratios even when the disease is rare. We use the term "distributional interaction" to refer to interaction that appears when using incidence odds ratios that does not appear, or appears to a lesser degree, when using risk ratios. The interpretational problems that arise from this discrepancy can have important implications in epidemiologic research. Therefore, quantification of the relationship between the interaction odds ratio and the interaction risk ratio is warranted.

In this paper, we provide a formula to quantify the differences between incidence odds ratios and risk ratios when they are used to estimate effect modification on a multiplicative scale. Using this formula, we examine the conditions under which these two estimates diverge. Furthermore, we expand this discussion to the implications of using incidence odds ratios to assess effect modification on an additive scale. Finally, we illustrate how distributional interaction arises and the problems that it causes using an example from the literature.

Whenever the risk of the outcome variable is non-negligible, distributional interaction is possible. This is true even when the disease is rare (e.g., disease risk is less than 5%). Therefore, when assessing interaction on either an additive or multiplicative scale, caution should be taken in interpreting interaction estimates based on incidence odds ratios.

## Introduction

It is well known that the incidence odds ratio approximates the risk ratio when the disease of interest is rare, but increasingly overestimates the risk ratio as the disease becomes more common. Therefore, it may not be surprising that odds ratios can give the impression of effect modification when none exists among the corresponding risk ratios. Despite this intuition, there is little formal discussion of this phenomenon in the epidemiologic literature. In what follows, we address this issue in an effort to enhance our understanding of the influence of effect measure selection on the interpretation of study results.

The risk ratio, which compares the probability of disease in the exposed and unexposed, is a conceptually appealing measure of effect. A risk ratio (RR) of 2, for example, is easily understood as a doubling of risk. This is in stark contrast to the interpretational obscurity of the incidence odds ratio (OR) [1]. When we use the term "incidence odds ratio," we are referring to the OR calculated from data on disease status obtained at the end of the observation period. This is the type of odds ratio calculated from fixed cohort studies and from case control studies that use cumulative sampling methods (see endnote 1). Despite this interpretational problem, the OR is frequently used because of its appealing statistical properties. The interpretational hurdle is overcome by treating the OR as if it were a RR, by understanding an OR of 2, for example, as a doubling of risk (see endnote 2). This interpretational license is innocuous as long as the RR is reasonably small and the probability of disease in the unexposed is reasonably low. Several formulas have been developed to quantify the discrepancies between the RR and the OR and thus prevent misinterpretation [2-5]. The general principle that the OR approximates the RR under the rare disease assumption is well known.

The implications of the "rare disease assumption" for the interpretation of interaction (i.e., effect modification) on the multiplicative or additive scale when using ORs have received less attention. However, interpreting the OR as if it were an RR can be particularly misleading when examining effect modification [6], as illustrated in the hypothetical cohort study presented in Table 1. For simplicity, we constructed this and all our examples with no confounding and no sampling or measurement error.

**Table 1:** Illustrative example: a hypothetical cohort study of the relationship between low aspirin intake and polyps among individuals with and without the high-risk COX-2 genotype

	Individuals with high-risk COX-2 genotype		Individuals without high-risk COX-2 genotype	
	Polyps	No Polyps	Polyps	No Polyps
Low aspirin intake	75	25	30	70
High aspirin intake	25	75	10	90
**Risk Ratio**	**3.0**		**3.0**	
**Odds Ratio**	**9.0**		**3.9**	

In this illustrative example, the RR for the relationship between low aspirin intake and the presence of polyps is the same for individuals with and without the high-risk COX-2 genotype. That is, the RRs do not provide evidence for effect modification on a multiplicative scale. Among those without the high-risk genotype, for whom the incidence of the disease is low, the OR is a close approximation of the RR. Among those with the high-risk genotype, however, where the disease is common, the OR is not a good approximation of the RR. This leads to considerable heterogeneity of the OR that would be interpreted as evidence of effect modification. Morabia et al. referred to this discrepancy as "the interaction fallacy" [7]. However, as Walter notes, this is not a statistical fallacy, since there is indeed effect modification of the OR [8]. Nonetheless, there is an interpretational fallacy if the conclusion reached based on these results is that low aspirin intake increases the risk (on a multiplicative scale) of polyps more for individuals with the high-risk COX-2 genotype than for individuals without the high-risk genotype. The effect of the risk factor appears to differ between strata due solely to a difference in the disease risk between strata. That is, this artifact appears because the disease is differentially distributed across strata. We therefore refer to this phenomenon as "distributional interaction" (see endnote 3).

To avoid this interpretational problem, we need to know the conditions under which effect modification of the OR is likely to approximate effect modification of the RR and when it is not. This understanding necessitates clarification of the elements that cause this discrepancy. Although Morabia et al. reported the existence of this phenomenon, these elements have not been previously explicated in the literature. Furthermore, this discussion has not been extended to additive interaction. To address this problem, we 1) provide a quantification of the relationship between effect modification of the RR and the OR on both multiplicative and additive scales, 2) examine the sensitivity of these relationships to variations in their determinants, and 3) use an example from the literature to highlight the causes and consequences of distributional interaction on either the multiplicative or additive scale.

## Analysis

Multiplicative interaction is commonly assessed by comparing the relative effect estimates for one risk factor across strata of another risk factor. Heterogeneity of the effect across strata is evidence of interaction. Here, we will use an equivalent method of assessing multiplicative interaction that we find more conceptually meaningful. This notion of interaction refers to the extent to which the joint effect of the two risk factors on disease differs from the independent effects of both factors. The joint effect is the effect of the presence of both factors on disease. Each factor's independent effect is its effect in the absence of the other factor. A comparison between the joint effect and the *product *of the independent effects provides a measure of multiplicative interaction. This calculation of multiplicative interaction (i.e. the ratio of the joint effect to the product of the independent effects) using RRs is shown below.

Throughout this paper, we refer to an interaction between a genetic and an environmental factor, as in the example of COX-2 genotype and aspirin in causing polyps described above. This discussion could be extrapolated to an interaction between any two factors (i.e. two genetic factors, two environmental factors, etc.).

### Notation

Here, G represents the genetic factor, E represents the environmental factor and D represents the disease. All three factors are dichotomous. For these factors, upper-case letters indicate the presence of the factor and lower-case letters indicate the absence of the factor. Assuming a multiplicative scale, the causal effect of the G-E interaction on D measured using RRs will be referred to as GxE_RR_. Similarly, the causal effect of the G-E interaction on D measured using ORs will be referred to as the GxE_OR_. The baseline risk of disease is represented by p(D|ge). This refers to the probability of disease [p(D)] among those who have neither the genetic nor the environmental risk factor.

### Assessment of Multiplicative Interaction

**Figure 1:**
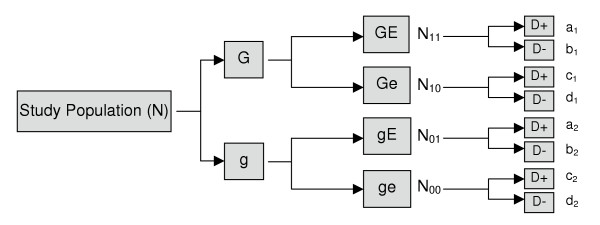
Schematic representation of study cohort

Population N is a population that underlies a hypothetical cohort study. The members of this population are categorized according to their exposure to G and E and followed for incident disease. Because these two exposures are dichotomous, individuals are classified according to four exposure categories. This hypothetical cohort study is illustrated in figure 1.

In this cohort study, those exposed to both G and E are represented by N_11_, N_10 _represents the sub-population that is exposed to G and unexposed to E, N_01 _corresponds to the sub-population that is unexposed to G and exposed to E, and N_00 _corresponds to the sub-population that is unexposed to both factors. These individuals can be further classified according to their D status. The cells labeled a_1 _to d_1 _represent those exposed to G and the cells labeled a_2 _to d_2 _represent those unexposed to G.

**Figure 2:**
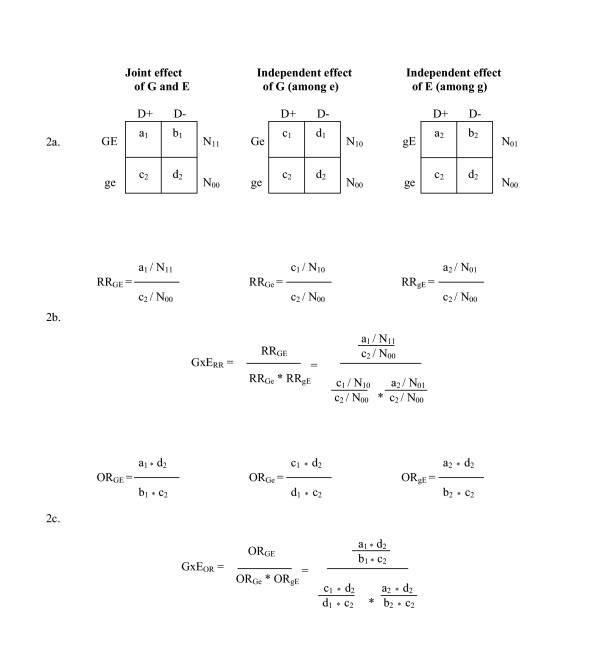
Assessment of multiplicative interaction using risk ratios and odds ratios.

To demonstrate the calculation of the interaction between G and E using RRs, we have organized the cells a_1 _to d_2 _into the two-by-two tables presented in figure 2a. The joint and independent effects of G and E, and the calculation of the gene-environment interaction are shown in figure 2b. The baseline risk of disease, or the risk of disease attributable to factors other than G and/or E, is represented by (c_2 _/ N_00_). The RR for the joint effect of G and E (RR_GE_) is calculated by comparing the risk of disease among those who are exposed to both G and E to the baseline risk. The RR for the independent effect of G (RR_Ge_) compares the risk of disease among those who are exposed to G and unexposed to E to the baseline risk. Similarly, the RR for the independent effect of E (RR_gE_) compares the risk of disease among those who are unexposed to G and exposed to E to the baseline risk. Using these components, the gene-environment interaction based on RRs (GxE_RR_) is measured by the ratio of the joint RR to the product of the independent RRs.

Using the same notation and the two-by two tables shown in parts a and b of figure 2, the joint and independent effects of G and E measured by ORs can also be derived (see figure 2c). Just as the joint and independent effects measured by RRs are used to calculate the GxE_RR_, the joint and independent effects measured by ORs are used to calculate the GxE_OR_.

### Relationship between the GxE_RR _and the GxE_OR_

Several algebraically equivalent formulations of the relationship between the OR and the RR have been discussed in the literature [2-5]. This relationship can be expressed in terms of two components – the size of the RR and the magnitude of the baseline risk, i.e. the risk of the disease in those who are unexposed (equation1) [4].



Similarly, the relationship between the GxE_OR _and the GxE_RR _depends in part on the magnitude of the GxE_RR _and the baseline risk of disease. Equation 1 can be used to calculate the OR for each of the three components of the GxE_RR_, resulting in the OR_GE_, OR_Ge _and OR_gE_. Consequently, the relationship between the GxE_RR _and the GxE_OR _can be described using equation 2, shown below.



A rearrangement of this formula is shown below in equation 3.



Equation 3 illustrates the factors that determine the relationship between the GxE_RR _and the GxE_OR_: the size of the GxE_RR_, the magnitude of the baseline risk of disease, and the strength of the independent effects.

For illustration, the data for the relationship between aspirin, COX-2 genotype and polyps shown in table 1 can be rearranged according to the three tables depicted in figure 2. In this example, the GxE_RR _is 1 and the baseline risk of disease (i.e. the probability of disease among individuals without the high-risk genotype and with high aspirin intake) is 10%. Additionally, the RR for the independent effect of low aspirin intake (among individuals without the high-risk genotype) is 3 (RR_Ge _= 3), the RR for the independent effect of the high-risk COX-2 genotype (among those with high aspirin intake) is 2.5 (RR_gE _= 2.5), and the RR for the joint effect of low aspirin intake and the high-risk COX-2 genotype is 7.5 (RR_GE _= 7.5). When these four elements are entered into equation 3, the GxE_OR _is 2.3.

### Effect of Disease Risk on the Relationship between GxE_OR _and GxE_RR_

In the absence of independent effects, equation 2 reduces to equation 1. That is, the relationship between the GxE_OR _and the GxE_RR _is equivalent to the relationship between the OR_GE _and the RR_GE. _This relationship can be fully expressed in terms of the RR_GE _and the baseline risk. When the RR is 1, the OR is also 1, regardless of the baseline risk of disease. Similarly, when the GxE_RR _is 1 and there are no independent effects, the GxE_OR _is also 1, regardless of the baseline risk of disease. In the absence of independent effects, as the magnitude of the GxE_RR _increases, the GxE_OR _diverges from the GxE_RR_. At a constant GxE_RR_, the divergence increases as the baseline risk of disease increases and becomes appreciable as the baseline risk of disease approaches 10%. Thus when there are no independent effects, the relationship between the GxE_OR _and the GxE_RR _is precisely the same as the relationship between the OR and the RR used to estimate main effects.

**Figure 3:**
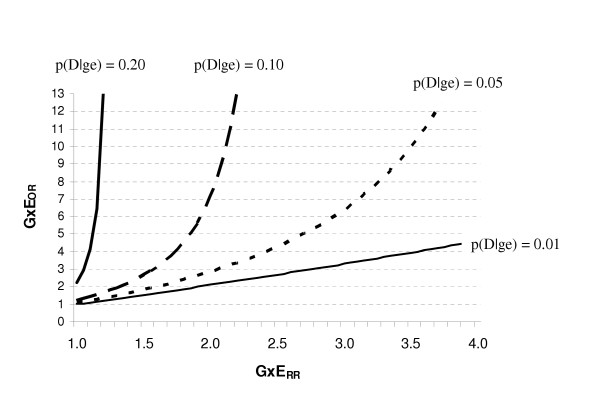
The effects of the baseline risk of disease [p(D|ge)] and the magnitude of the multiplicative interaction estimate based on risk ratios (GxE_RR_) on the multiplicative interaction estimate based on odds ratios (GxE_OR_) when the RR for each independent effect is 2 (RR_Ge _= RR_gE _= 2).

When there are independent effects, the relationship between the GxE_OR _and GxE_RR _is more complex. Figure 3 illustrates the situation in which the RR for each independent effect is 2. As shown in this graph, the GxE_OR _is a good approximation of the GxE_RR _when the disease is very rare [p(D|ge) = 1%], regardless of the magnitude of the GxE_RR_. However, as the baseline risk of disease approaches 5%, the GxE_OR _begins to appreciably overestimate the GxE_RR_. In general, the larger the baseline risk of disease, the worse the approximation, even at moderate strengths of interaction. In fact, when the risk of the disease is 20%, the GxE_OR _indicates a strong interaction when the RRs do not provide evidence for interaction.

**Figure 4:**
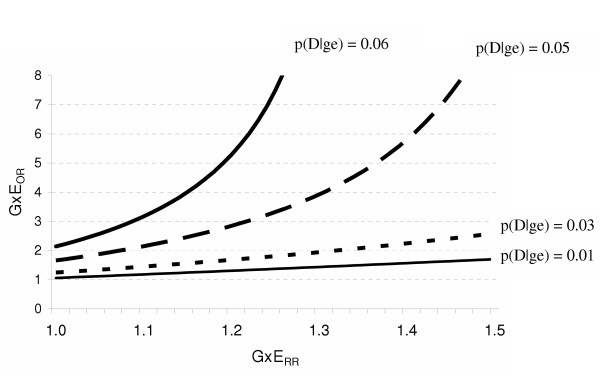
The effects of the baseline risk of disease [p(D|ge)] and the magnitude of the multiplicative interaction estimate based on risk ratios (GxE_RR_) on the multiplicative interaction estimate based on odds ratios (GxE_OR_) when the RR for the independent effect of G is 6 (RR_Ge _= 6) and the RR for the independent effect of E is 2 (RR_gE _= 2).

Figure 4 highlights the situations in which a researcher would reasonably conclude that there is no evidence for a substantial multiplicative interaction based on RRs (i.e. the GxE_RR _ranges from 1.0 to 1.5). In this circumstance, the GxE_OR _is a fair approximation to the GxE_RR _when the disease is very rare [p(D|ge) = 1%]. That is, a researcher using the GxE_OR _would appropriately conclude that there is no evidence for a multiplicative interaction. Here, as the baseline risk of disease approaches 3%, the GxE_OR _begins to appreciably overestimate the GxE_RR_. For example, when the GxE_RR _is 1.3, the GxE_OR _is 2. Although this is not a large overestimation it has important implications for interpretation. A researcher may not find a GxE_RR _of 1.3 indicative of a substantial multiplicative interaction, but may conclude otherwise when the estimate is a 2. Again, the larger the baseline risk of disease, the worse the approximation. When at least one of the independent effects is strong and the risk of the disease is greater than 1%, the GxE_OR _is a poor approximation to the GxE_RR _even when the GxE_RR _is weak.

### Implications for Assessment of Additive Interaction

The analyses we have shown so far indicate that using ORs to assess multiplicative interaction can lead to interpretational difficulties. We have focused on multiplicative interaction because this is the most common scale on which interaction is assessed when ORs are used as a measure of effect. However, the appropriate scale on which to assess interaction has been a controversial topic in epidemiology. Darroch [9] and Rothman and Greenland [10] have mounted a persuasive argument for the use of the additive scale to assess the presence of synergistic effects. As they note, additive interaction can be expressed in terms of RRs through the interaction contrast ratio. In Equations 4 and 5 below, we refer to the interaction contrast ratio calculated with RRs and ORs as the ICRR and ICOR, respectively.

Eq.4 ICRR = RR_GE _- RR_Ge _- RR_gE _+ 1

Eq.5 ICOR = OR_GE _- OR_Ge _- OR_eE _+ 1



**Figure 5:**
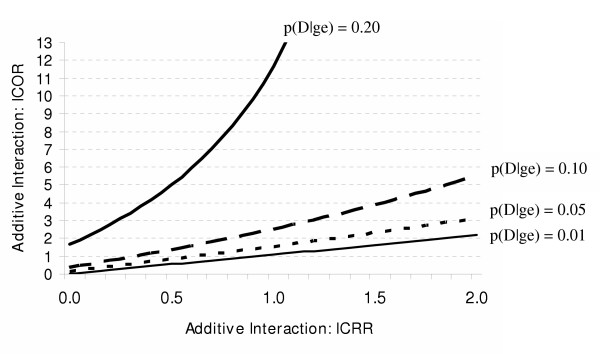
The effects of the baseline risk of disease [p(D|ge)] and the magnitude of the additive interaction estimate based on risk ratios (ICRR) on the additive interaction estimate based on odds ratios (ICOR) when the RR for each independent effect is 2 (RR_Ge _= RR_gE _= 2).

**Figure 6:**
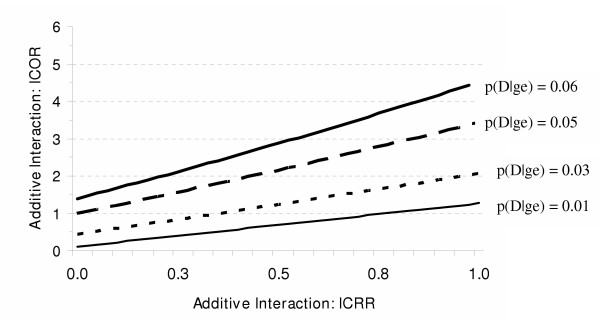
The effects of the baseline risk of disease [p(D|ge)] and the magnitude of the additive interaction estimate based on risk ratios (ICRR) on the additive interaction estimate based on odds ratios (ICOR) when the RR for the independent effect of G is 6 (RR_Ge _= 6) and the RR for the independent effect of E is 2 (RR_gE _= 2).

As seen in the assessment of multiplicative interaction (equation 2), the three component ORs of the ICOR formula can be replaced with the corresponding RR-OR equivalence formula (equation 5). Consequently, the factors that determine the relationship between the GxE_RR _and the GxE_OR _are the same factors that determine the relationship between the ICRR and the ICOR. Again, these factors are the magnitude of the GxE_RR_, the baseline risk of disease and the strength of the independent effects. The ICOR can result in the appearance of additive interaction when none would be observed using the ICRR, or can substantially overestimate the magnitude of additive interaction given by the ICRR (see figures 5 and 6).

For illustration, we use the scenario depicted in figure 6, where the RR_Ge _is 6, the RR_gE _is 2, and the baseline risk of disease is 6%. In the absence of additive interaction, the RR_GE _would be 7. The ICRR would reflect this absence of additive interaction (equation 6).

Eq. 6 ICRR = RR_GE _- RR_Ge _- RR_gE _+ 1 = 7 - 6 - 2 + 1 = 0

However, as shown in equation 7, the ICOR would not equal 0 and would give the appearance of interaction.

Eq. 7 ICOR = OR_GE _- OR_Ge _- OR_gE _+ 1 = 11.3 - 8.8 - 2.1 + 1.0 = 1.4

In this instance, one would reasonably interpret the results from equation 7 as evidence of additive interaction even though the same data using RRs (equation 6) would give no indication of additive interaction. Therefore, the problem of the OR overestimating the RR applies to interaction on the additive scale, in addition to the multiplicative scale.

### An example of distributional interaction from the literature

The discrepancy between incidence odds ratio- and risk ratio-based estimates of interaction was noted by Li et al. in an occupational cohort study of mutant p53 positivity, a biomarker of genetic damage [11]. The authors reported estimates of the multiplicative interaction between a polymorphism in a DNA repair gene, XRCC1, and exposure to vinyl chloride. They found a multiplicative interaction estimate based on ORs of 2.91. However, the interaction estimate based on RRs was only 1.24 (see table 2). In a reexamination of their data, we found that the ORs substantially overestimated the additive interaction based on risk ratios as well. The ICRR was 0.96 (RR_GE _= 3.20, RR_Ge _= 1.82, RR_gE _= 1.42) and the ICOR was 8.85 (OR_GE _= 12.00, OR_Ge _= 2.50, OR_gE _= 1.65). In this circumstance, the overestimation can be attributed to the high baseline risk of the outcome among this cohort of vinyl chloride workers, 25%. These data highlight the potential for distributional interaction.

**Table 2:** Example from Li et al: "A common polymorphism in XRCC1 as a biomarker of susceptibility for chemically induced genetic damage" [11]

	Individuals with XRCC1 high-risk genotype (*Gln – Gln*)		Individuals with XRCC1 low-risk genotype (*Arg – Arg*)	
	p53+	p53-	p53+	p53-
High vinyl chloride exposure (>4000 ppm-yrs)	8	2	11	20
Low vinyl chloride exposure (≤1000 ppm-yrs)	5	6	6	18
**Risk Ratio**	**1.76**		**1.42**	
**Odds Ratio**	**4.80**		**1.65**	

## Conclusion

The incidence odds ratio is a very convenient measure of effect with many appealing statistical properties including estimability in a case-control study. However, when assessing interaction, as when assessing main effects, interpreting the incidence odds ratio as if it were a risk ratio can be misleading. In particular, when the interaction based on risk ratios is reasonably high and the probability of disease among the unexposed is non-negligible, the interaction based on incidence odds ratios may diverge appreciably from the interaction based on risk ratios. Even when the disease is rare (e.g.,. disease risk is less than 5%), the incidence odds ratios can give the appearance of interaction, on either the additive or multiplicative scale, when the risk ratios would indicate the absence of interaction.

In most situations where incidence odds ratios are used to assess interaction, the corresponding interaction based on risk ratios, the baseline risk of disease, and the strength of the independent effects are unknown. Therefore, the divergence of the interaction based on incidence odds ratios from the interaction based on risk ratios is unknown. The GxE_RR _can be estimated using equation A1 (Appendix 1) if the odds ratios for the joint and independents effects (OR_GE_, OR_Ge_, OR_gE_) are known and a valid estimate of the baseline risk of disease [p(D|ge)] is available. However, even when these factors can be estimated, seemingly small errors in these estimates at critical thresholds can create a false sense of security. For example, there are circumstances as illustrated above, where a difference of as little as 2 percentage points in the probability of disease differentiates a valid from an invalid assessment of interaction using incidence odds ratios. Such small differences can easily be the result of measurement or sampling error.

This paper provides guidelines for assessing the conditions under which there may be interpretational difficulties when using incidence odds ratios to assess interaction. The first guideline, that caution must be used when the outcome under investigation is relatively common, is consistent with the well known "rare disease assumption". Based on these analyses, there are many situations where the baseline risks are likely to be high enough and the interactions based on risk ratios strong enough to warrant caution. For example, in epidemiologic studies of psychiatric disorders, disease risk is often above 10%. In cancer epidemiology, when intermediate endpoints or biomarkers are of interest, outcome risks are often in the range of 30 to 50%. Under such circumstances, the equations and figures provided in this paper could be used to estimate the divergence between the interaction estimates based on incidence odds ratios and the underlying risk ratios. Regardless of design, in studies of interaction for common outcomes, incidence odds ratios should be interpreted with caution. The second guideline is less in line with conventional epidemiologic wisdom. Even when the outcome is rare, there are circumstances in which the incidence odds ratios can still be quite misleading when assessing interaction (e.g. the independent effect of the gene is strong).

When assessing either multiplicative or additive interaction using incidence odds ratios, consideration should always be given to the validity of interpreting the odds ratios as risk ratios. The danger of distributional interaction, the appearance of interaction when using incidence odds ratios that would not exist if risk ratios were used or the exaggeration of the magnitude of the interaction based on risk ratios, should always be considered.

## Abbreviations

RR, risk ratio • OR, incidence odds ratio • G, genetic factor • E, environmental factor • D, disease • GxE_RR_, multiplicative gene-environment interaction estimate based on risk ratios • GxE_OR_, multiplicative gene-environment interaction estimate based on odds ratios • p(D), probability of disease • p(D|G-E-), probability of disease among people without G and E (baseline risk of disease) • RR_GE_, risk ratio for joint effect of G and E • RR_Ge_, risk ratio for independent effect of G • RR_gE_, risk ratio for independent effect of E • OR_GE_, odds ratio for joint effect of G and E • OR_Ge_, odds ratio for independent effect of G • OR_gE_, odds ratio for independent effect of E • ICRR, interaction contrast ratio calculated with risk ratios • ICOR, interaction contrast ratio calculated with odds ratios

## Endnotes

1: This type of odds ratio might also be called the cumulative incidence odds ratio, risk relative odds ratio, probability relative odds ratio or Cornfield's odds ratio [10,12]. The discussion in this paper only applies to this type of odds ratio. It does not apply to odds ratios that result from case-control studies in which controls are sampled from everyone at risk (as in a case-cohort design). In such designs, the odds are a constant multiple of the risk, and their ratio is identical to the RR regardless of the rarity of the disease. Similarly, the OR calculated from case-control studies using incidence density sampling is equivalent to the rate ratio regardless of disease rarity.

2: Treating the OR as an approximation of the rate ratio, rather than the risk ratio may be preferable in many contexts. But the interpretation of the OR as the risk ratio is most common in practice.

3: We were introduced to the term "distributional interaction" by Dr. Stephen Ng when he was teaching epidemiology at Columbia University. However, we have not been able to locate any articles or books that use the term, although we suspect that it may derive from Miettinen's discussion of collapsibility. We prefer it to "interaction fallacy" because a) it avoids the implication that the interaction is statistically incorrect and b) it is descriptive of the source of the interaction. It arises from the different distribution of disease across strata.

## Appendix 1

If the odds ratios for the joint and independents effects (OR_GE_, OR_Ge_, OR_gE_) are known and data on the baseline risk of disease [p(D|ge)] are available, the GxE_RR _may be estimated. Below, we provide a reformulation of equation 2 to estimate the GxE_RR _based upon these inputs (equation A1).


